# Strain Engineering of Octahedral Rotations and Physical Properties of SrRuO_3_ Films

**DOI:** 10.1038/srep10245

**Published:** 2015-05-28

**Authors:** Wenlai Lu, Wendong Song, Ping Yang, Jun Ding, Gan Moog Chow, Jingsheng Chen

**Affiliations:** 1 Department of Materials Science and Engineering, National University of Singapore, Singapore 117576; 2 Data Storage Institute, A*STAR (Agency for Science, Technology and Research), DSI Building, 5 Engineering Drive 1, Singapore 117608; 3 Singapore Synchrotron Light Source (SSLS), National University of Singapore, 5 Research Link, Singapore 117603

## Abstract

Strain engineering is an effective way to modify functional properties of thin films. Recently, the importance of octahedral rotations in pervoskite films has been recognized in discovering and designing new functional phases. Octahedral behavior of SrRuO_3_ film as a popular electrode in heterostructured devices is of particular interest for its probable interfacial coupling of octahedra with the functional overlayers. Here we report the strain engineering of octahedral rotations and physical properties that has been achieved in SrRuO_3_ films in response to the substrate-induced misfit strains of almost the same amplitude but of opposite signs. It shows that the compressively strained film on NdGaO_3_ substrate displays a rotation pattern of a tetragonal phase whilst the tensilely strained film on KTaO_3_ substrate has the rotation pattern of the bulk orthorhombic SrRuO_3_ phase. In addition, the compressively strained film displays a perpendicular magnetic anisotropy while the tensilely strained film has the magnetic easy axis lying in the film plane. The results show the prospect of strain engineered octahedral architecture in producing desired property and novel functionality in the class of perovskite material.

Octahedral behavior in perovskite materials has attracted broad attention due to its critical role in determining the functionalities of transition metal oxides and their applications. Interfacial octahedral coupling is of particular interest since it may produce some unexpected properties that are absent in the bulk form. Typical examples are large and stable dielectric constant achieved in ferroelectric/paraelectric PbTiO_3_/SrTiO_3_ superlattice over a wide range of temperature^1^ and strongly modified ferromagnetism in antiferromagnetic/diamagnetic LaMnO_3_/SrTiO_3_ superlattice^2^. SrRuO_3_ (SRO) film as a popular electrode is used in various oxide devices. It is therefore reasonable to expect that the functionalities of the overlayer and the performance of the heterostructure device are highly dependent on the octahedral behavior of the SRO via interfacial octahedral coupling.

Bulk SRO adopts a GdFeO_3_-type orthorhombic structure^3^ at room temperature in which the RuO_6_ octahedra are rotated in opposite directions by equivalent magnitude about [100] and [010] directions, and in the same direction along [001]. Such rotation pattern is denoted by **a**^**−**^**a**^**−**^**c**^**+**^in the Glazer notation^4^. Structural phase transitions in bulk SRO are associated with the RuO_6_ octahedral rotations. As the temperature is raised from room temperature to 547 °C, the orthorhombic phase of bulk SRO with **a**^**−**^**a**^**−**^**c**^**+**^rotation pattern transits to the tetragonal phase with **a**^**0**^**a**^**0**^**c**^**−**^ pattern where the RuO_6_ octahedra are rotated only about [001] direction and, finally, high-symmetry cubic phase is stabilized above 677 °C with no rotations of octahedra at all, denoted by **a**^**0**^**a**^**0**^**a**^0^ 5.

The structural phase transition in strained epitaxial SRO films has been widely investigated. It was reported that in strained SRO film on SrTiO_3_ (STO) substrate, the transition temperature of the orthorhombic to cubic phase is lowered compared to that of the bulk SRO, suggesting that the substrate-induced strain has a strong influence on the structural phase transitions associated with the RuO_6_ octahedral rotations^6^,^7^. Recently, it has been shown that SRO film on (001) STO substrate transits from the monoclinic to tetragonal phase with either introducing oxygen vacancies, reducing the film thickness^8^, or imposing tensile strain, i.e.,+0.7% on (110) DyScO_3_ (DSO)^9^ and+1.0% on (110) GdScO_3_(GSO)^10^. Despite the critical role of the substrate-induced strain in the structural phase transitions and octahedral behaviors, how the RuO_6_ octahedra respond to the compressive and tensile strain in the perovskite films has not yet been fully understood.

In this study, two substrates – (110) NdGaO_3_ (NGO) and (001) KTaO_3_ (KTO) - that have the lattice mismatch of almost the same magnitude but of opposite sign were chosen to grow the SRO films. The film underwent compressive strain of −1.7% when grown on (110) NGO substrate and tensile strain of+1.5% when grown on (001) KTO substrates. We found that the compressive and tensile strains showed different effects on the octahedral rotation of SRO. The schematic drawing of the effects of various strains on octahedral rotation of SRO films is shown in Fig. 1. An enhanced octahedral rotation about the out-of-plane axis would result from the compressive strain (see Fig. 1(a)), leading to a distorted orthorhombic lattice of SRO film on (001) STO substrate^9^,^11^ and perpendicularly oriented tetragonal SRO phase as found in SRO film on (110) NGO substrate. On the other hand, the tensile strain has the effect of stretching the corner connected octahedral network, leading to the diminished rotations of octahedra about the out-of-plane direction (Fig. 1(c)), as has been reported of SRO film on GSO substrate^10^. Further increasing the tensile strain however, leads to a relaxed bulk-like SRO phase, which is the case of SRO film grown on KTO substrate (Fig. 1(d)). The octahedral response to different strains in SRO films has emerged as a critical step towards a better understanding of octahedral behavior in other strained perovskite films, and may allow for the manipulation of octahedral-behavior-related functionalities by strain engineering.

## Results and Discussion

Figure 2(a) shows the reciprocal space L-scans (equivalent to *θ*-2*θ* coupled scans of x-ray diffraction (XRD)) for the SRO films grown on NGO and KTO substrates respectively. We only observed the (00*l*) type diffraction peaks from the film layer and the substrate over the whole scan range, confirming the epitaxial growth of the film. In addition, the SRO (00*l*) peaks locate at the left side of the NGO (00*l*) peak and at the right side of the KTO (00*l*) peak, indicating the compressive strain and tensile strain states respectively in the films. The out-of-plane lattice parameter of SRO films calculated from the (002) peak was calculated to be *c* = 4.025 Å for SRO/NGO and *c* = 3.911 Å for SRO/KTO respectively.

Figure 2(b) shows the reciprocal space mappings (RSMs) around NGO {103} reflections (top) and KTO {103} reflections (bottom) which can be used to obtain the in-plane lattice parameters. Note that NGO has different L values of (013) and (0-13) reflections, indicating an oblique angle between *b* and *c*, consistent with the orthorhombic symmetry of NGO material^12^,^13^. Consider the position of the SRO {103} reciprocal lattice point in the {103} mapping. For SRO/NGO, the film is fully strained, as inferred from the same H value of the SRO and NGO reciprocal lattice point. The unit cell of SRO shrinks in the film plane (from 3.930 Å to 3.857 Å) and is elongated along z-axis (from 3.930 Å to 4.025 Å) as a consequence of the compressive strain. For SRO/KTO, on the contrary, the unit cell expands in the film plane (from 3.930 Å to 3.950 Å) as a consequence of the tensile strain imposed by the KTO substrate (*a* = 3.989 Å), and shrinks along z-axis (from 3.930 Å to 3.911 Å) due to conservation of unit cell volume. Despite almost the same lattice mismatch, the SRO is fully strained on NGO substrate (compressive strain) whereas partially relaxed on KTO substrate (tensile strain). It seems that the SRO film is more reluctant to be fully strained when undergoes tensile strain than compressive strain. This phenomenon is correlated to the different octahedral response to compressive and tensile strains and will be explained in more detail later.

In order to investigate the octahedral behavior in compressive strained and tensile strained SRO films, half-integer reflections were examined to identify the octahedral rotation patterns (or octahedral tilt systems) of both samples. According to Glazer^4^, peaks of the type ^1^/_2_{*ooo*} (*o* stands for odd integer) occur when the octahedra are rotated out-of-phase. On the other hand, when the octahedra are rotated in an in-phase manner, half-integer reflections of the type ^1^/_2_{*ooe*} (*e* stands for even integer) are produced^4^. However, these peaks are usually weak as a consequence of the small atomic form factor of oxygen ions. Therefore, high flux synchrotron x-ray sources were necessarily used in this experiment to obtain the half-integer peaks. The results of half-integer reflections of SRO films on NGO substrate and KTO substrate are shown in Fig. 3(a,b) respectively.

**Figure 1:**
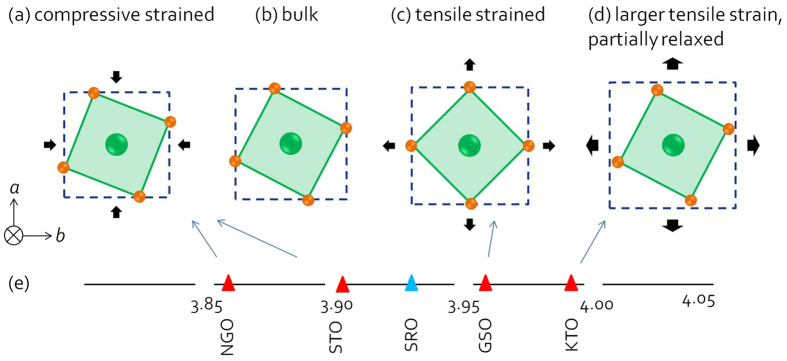
Schematic of the RuO_6_ octahedral rotation when the SRO is (**a**) compressive strained, (**b**) in bulk, (**c**) tensile strained or (**d**) partially relaxed. Ru atoms and O atoms are in green and orange respectively. Pseudocubic unit cell of the substrates is represented by blue squares. The relationship between pseudocubic lattice parameters of SRO and the substrates mentioned in this paper is shown in (**e**).

**Figure 2:**
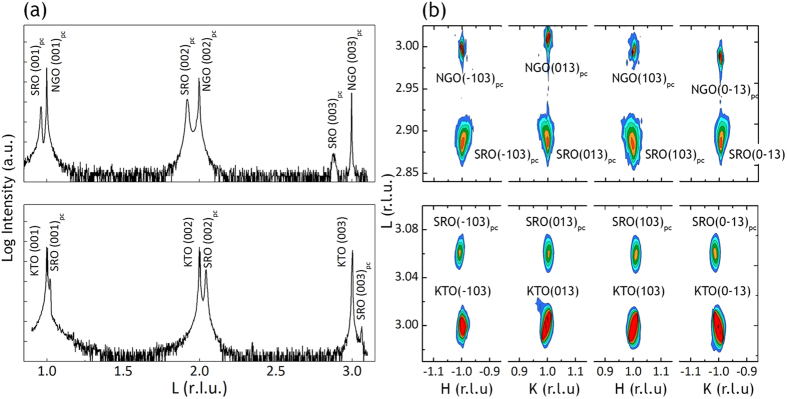
Reciprocal space L-scans and reciprocal space mappings of SRO films. (**a**) Reciprocal space L-scans of SRO films deposited on NGO substrate (top) and KTO substrate (bottom) respectively. (**b**) Reciprocal space mappings (RSMs) around {103} reflections of SRO films on NGO substrate (top panel) and KTO substrate (bottom panel) respectively.

**Figure 3:**
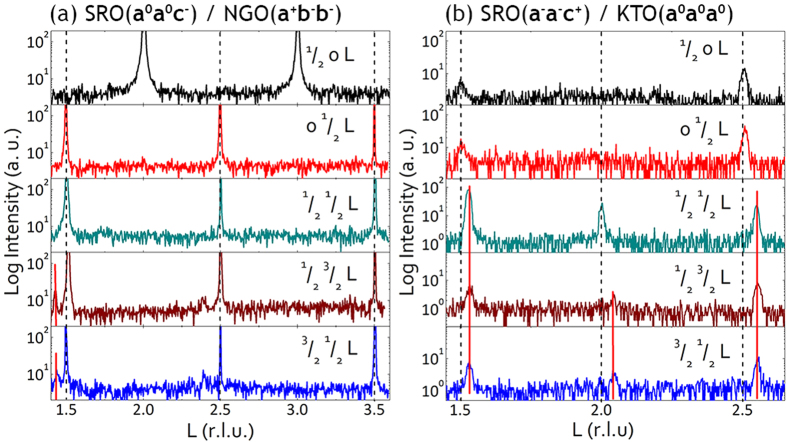
Half-integer reflections of SRO film deposited on NGO and KTO substrate. (**a**) SRO on NGO displays a rotation pattern of **a**^**0**^**a**^**0**^**c**^**-**^ corresponding to a tetragonal phase. (**b**) SRO on KTO substrate shows a tilt system of **a**^**−**^**a**^**−**^**c**^**+**^ indicative of an orthorhombic phase.

In Fig. 3(a), sharp peaks with high-intensity are observed. However, the peak positions (at exactly ^3^/_2_, 2, ^5^/_2_, 3 and ^7^/_2_) and the narrow width of the peaks demonstrate that these peaks (indicated by black dashed lines) should be assigned to the NGO substrate instead of the film. From the appearance and absence of certain peaks, the rotation pattern of **a**^**+**^**b**^**−**^**b**^**−**^ is immediately suggested for the NGO substrate, which is consistent with the literature^14^. In addition to the peaks arising from the substrate, peaks that correspond to SRO film are also observed. These peaks, as indicated by red lines in Fig. 3(a), locate just at the left side of the substrate’s peaks due to their larger lattice constant than that of substrate. Since the only appeared reflections ^1^/_2_^3^/_2_^3^/_2_ and ^3^/_2_^1^/_2_^3^/_2_ are of the ^1^/_2_ (*ooo*) type, the tilt system **a**^**0**^**a**^**0**^**c**^**−**^ is assigned with the only octahedral rotations about the perpendicular direction. Note the absence of ^1^/_2_^1^/_2_^3^/_2_ reflection indicates the absence of **a**^**−**^ or **b**^**−**^ tilt. From the half-integer reflection results, we infer that the rotations of octahedra in compressively strained SRO film are enhanced than its bulk counterpart about the out-of-plane direction to accommodate the compressive strain (see Fig. 1(a)). The space group is *F4*/*mmc* and the crystal symmetry is tetragonal for the compressive strained SRO film on NGO substrate.

As for the SRO film grown on KTO substrate, half-integer peaks were observed at the right side of L = ^3^/_2_ and ^5^/_2_, arising from the SRO film as indicated by red lines in Fig. 3(b). Among them, the most notable one is the ^1^/_2_^1^/_2_^3^/_2_ peak that corresponds to **a**^**−**^ and **b**^**−**^ tilts, which are the out-of-phase rotations about in-plane directions. However, it is difficult to determine if there is **c**^**−**^ tilt or not: the peak ^1^/_2_^3^/_2_^3^/_2_ may arise from **b**^**−**^ only, or the combination of **b**^**−**^ and **c**^**−**^; similarly, the peak ^3^/_2_^1^/_2_^3^/_2_ may arise from **a**^**−**^ only, or the combination of **a**^**−**^ and **c**^**−**^. Considering that the intensity of ^1^/_2_^3^/_2_^3^/_2_, ^3^/_2_^1^/_2_^3^/_2_ peaks is almost one order of magnitude lower than that of the ^1^/_2_^1^/_2_^3^/_2_ peak, the **c**^**−**^ tilt, even if existent, can be negligible compared to **a**^**−**^ and **b**^**−**^ tilt. On the other hand, the ^1^/_2_^3^/_2_ 2 and ^3^/_2_^1^/_2_ 2 peaks, although weak, can still be seen as a consequence of **c**^**+**^tilt. The existence of **c**^**+**^tilt further excludes the possibility of **c**^**−**^ tilt. Consequently, the tilt system, most probably **a**^**−**^**a**^**−**^**c**^**+**^, is suggested for the SRO film grown on KTO substrate. Note that although slightly altered, the rotation pattern of this SRO film is the same with that of the bulk SRO due to the partial relaxation of the tensilely strained film on KTO substrate (see Fig. 1(d)). In addition to the peaks arising from the shrunken out-of-plane lattice constant of the tensilely strained SRO film, there are also broad and weak peaks of the ^1^/_2_ {*eoo*} type at exactly ^3^/_2_ and ^5^_/2_ (indicated by black dashed lines in Fig. 3(b)). Considering the absence of octahedral rotations in KTO substrate (the tilt system is **a**^**0**^**a**^**0**^**a**^**0**^), the broad and weak peaks may probably result from the SRO interfacial layers that were being forced into an intermediate rotation state by the adjacent rotation-free substrate. It would also be possible that the distorted substrate top layers near the interface led to the weak and broad half diffraction peaks. In order to clarify the origin of the broad and weak peaks of the ^1^/_2_ {*eoo*} type at exactly ^3^/_2_, ^5^_/2_, high-resolution transmission electron microscopy (TEM) and reduced Fast Fourier Transform patterns (FFTs) are required.

**Figure 4:**
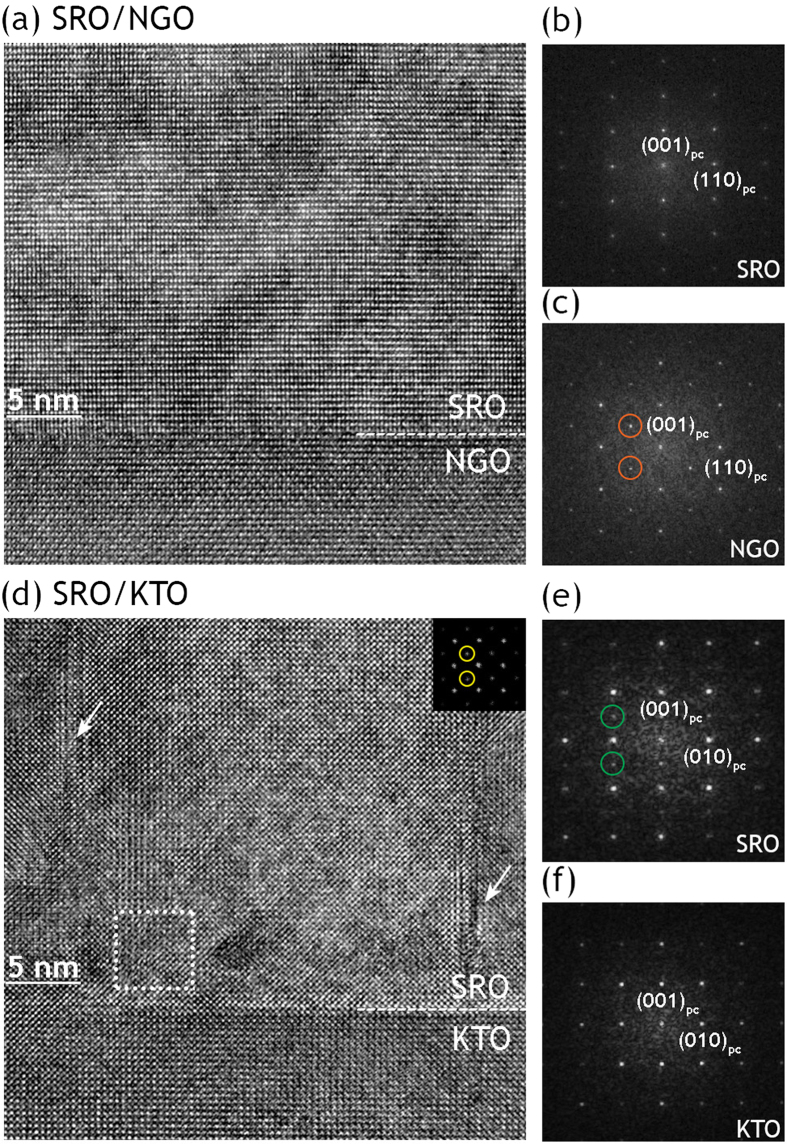
Cross-sectional HRTEM images and FFTs of SRO films on different substrates. (**a**) (**d**) Cross-sectional HRTEM images of SRO on (**a**) NGO and (**d**) KTO respectively. (**b**), (**c**), (**e**), (**f**) are FFTs of the films and substrates. Half-integer reflections ^1^/_2_{*ooo*} are circled in orange and ½ {*eeo*} reflections are circled in green. In (**d**), arrows indicate the location of dislocations and inset is the FFT of the selected area surrounded by square. The absence and presence of ^1^/_2_{*ooo*} type reflection in (**b**) and (**c**) indicates the absence of **b**^**−**^ tilt in SRO film and the presence of **b**^**−**^ tilt in the NGO substrate. Similarly, the existence of ^1^/_2_ {*eeo*} reflections in (**e**) suggests the **c**^**+**^ tilt in the SRO film deposited on KTO substrate.

In the following, the octahedral rotations in SRO films were examined by TEM and compared with the rotation pattern determined by XRD. The cross-sectional TEM image (Fig. 4(a)) shows that for SRO film on NGO substrate, there are no misfit dislocations at the interface, consistent with the coherent growth of SRO film on NGO substrate. In comparison, dislocations (indicated by arrows in Fig. 4(d)) were found in SRO/KTO sample both at the interface and away from the interface in order to accommodate the tensile strain, in accordance with the partial relaxation of the tensilely strained film on KTO substrate.

Figure 4(b,c,e,f) show the FFTs of the film and the substrate. In order to investigate the octahedral rotations in compressively strained SRO film on NGO substrate, we utilized the ^1^/_2_{*ooo*} type reflection with *h* = *k* as a finger print to differentiate the octahedral rotations for the NGO substrate (**a**^**+**^**b**^**−**^**b**^**−**^) and the SRO film (**a**^**0**^**a**^**0**^**c**^**−**^). Note that such type reflections could only be resulted from out-of-phase rotations about the in-plane axes (**a**^**−**^ or **b**^**−**^ tilt)^4^,^15^. In the FFTs of SRO/NGO the ^1^/_2_{*ooo*} type reflections with *h* = *k* (circled in orange in 4 (c)) were only seen for NGO substrate whereas they are absent for the SRO film. This suggests that the **b**^**−**^ tilt, which is present in the NGO, is corrupted in the SRO film about the two orthogonal in-plane axes. This is in agreement with the **a**^**0**^**a**^**0**^**c**^**−**^ tilt system determined from the half-integer reflections by XRD for the SRO/NGO film. In contrast, for tensilely strained SRO film, extra spots of the ^1^/_2_{*eeo*} type (circled in green in Fig. 4(e)) are visible in the FFTs of SRO/KTO in addition to the fundamental spots, indicative of the presence of **c**^**+**^ tilt (in-phase rotations of octahedra about the out-of-plane direction)^15^. This is consistent with the half-integer reflection measurement by XRD that the tensilely strained SRO film on KTO substrate has the **a**^**−**^**a**^**−**^**c**^**+**^ tilt system. Unlike the SRO film, no half-integer peaks of the ^1^/_2_{*eeo*} type are observed for the rotation-free KTO substrate. Finally, we took a close look at the SRO/KTO interface in order to determine the origin of the broad and weak half integer peaks (indicated by black dashed lines in Fig. 3(b)) of the ^1^/_2_{*eoo*} type observed by XRD. A small area of 5 nm × 5 nm was selected in the TEM image of SRO/KTO (Fig. 4(d)) both in the film as well as in the substrate top layers for the fourier transformation. It turns out that the ^1^/_2_{*eoo*} type diffraction spot (circled in yellow in the inset of Fig. 4(d)) was only observed at some part of the interfacial region of SRO film rather than KTO substrate. Consequently, it can be concluded that the KTO remains as-is, without any rotation of octahedra, while some part of the SRO interfacial layers was transformed into an intermediate octahedral rotation state by the substrate as an alternative to dislocations for the misfit relaxation.

On the basis of our experimental results, the structural determination by XRD study (including RSMs and half-integer reflection measurement) and TEM study agrees well – SRO film under −1.7% compressive strain is fully strained and favors the **a**^**0**^**a**^**0**^**c**^**−**^ tilt system of tetragonal phase, whilst SRO film under +1.5% tensile strain is partially relaxed and prefers the bulk-like phase with **a**^**−**^**a**^**−**^**c**^**+**^ rotation. In the following, how the magnetic properties and magnetotransport properties of the differently strained SRO films changes with the octahedral rotations is discussed.

**Figure 5:**
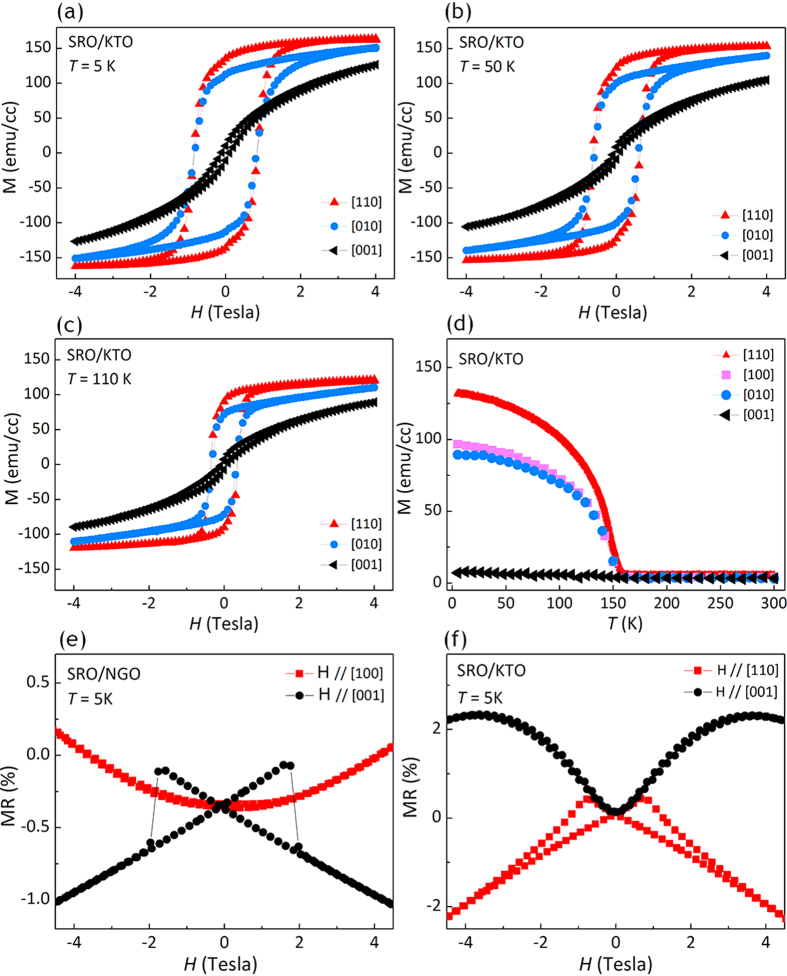
Magnetization measurement and field dependent magnetoresistance. (**a**) - (**c**) Magnetic hysteresis loops of SRO film on KTO substrate at (**a**) 5 K, (**b**) 50 K and (**c**) 110 K respectively. (**d**) Temperature dependence of magnetization along different crystalline directions of SRO/KTO. (**e**) - (**f**) MR versus magnetic field along in-plane direction (red curve) and out-of-plane direction (black curve) for SRO films on (**e**) NGO and (**f**) KTO substrate respectively.

The magnetic hysteresis loops of SRO film on KTO substrate at different temperatures are shown in Fig. 5(a–c). It was worth noting that regardless of temperature, the hysteresis loops along the in-plane [010] and [110] directions were more square-shaped whilst the out-of-plane hysteresis loop along [001] axis was line-like with a very small opening-up. The magnetic property was further examined by magnetization versus temperature measurement along different crystalline directions (Fig. 5(d)). The moment along the out-of-plane [001] direction is highly suppressed while the moment along the in-plane [110] direction is dramatically enhanced. It can be inferred from this result that the easy axis is probably in the film plane along [110] direction while the hard axis is along [001] direction for the SRO/KTO film, similar to that of the bulk SRO^16^. For the SRO film on NGO substrate however, the magnetic properties cannot be obtained straightforwardly from the SQUID measurement because of the paramagnetic of the NGO material. The magnetic moment from the NGO substrate would overlap the signal from the film considering the tremendous volume difference between substrate and film (the 0.5 mm thick substrate has a volume 10^4^ larger than the ~60 nm thick film). Consequently, the magnetoresistance MR = [ρ(*H*)- ρ(0)] / ρ(0) was utilized to investigate the magnetic anisotropy of both films, where ρ(*H*) and ρ(0) are the resistivity at a field *H* and at zero field, respectively. Figure 5(e) shows this data of SRO/NGO sample at 5 K. A strong hysteresis loop is observed which is related to the magnetization hysteresis. The peak in the MR-*H* loop corresponds to the coercive field and the point where the forward and backward sweeps overlap corresponds to the saturation field. In Fig. 5(e), a larger negative MR was observed when the field *H* is parallel to the out-of-plane [001] direction with a coercive field of 2 Tesla, probably due to the fact that the [001] direction is the easier axis of magnetization than the in-plane [100] direction. The MR behavior was unchanged when the sample was rotated by 90° (not shown here), indicating the equivalence of the two orthogonal directions within the film plane. In contrast to the SRO film on NGO substrate, the spins of SRO film on KTO substrate are more easily aligned within the film plane along [110] direction (as inferred from Fig. 5(a–d)) which accounts for the larger negative MR value along [110] direction compared to the out-of-plane [001] direction (Fig. 5(f)).

**Figure 6:**
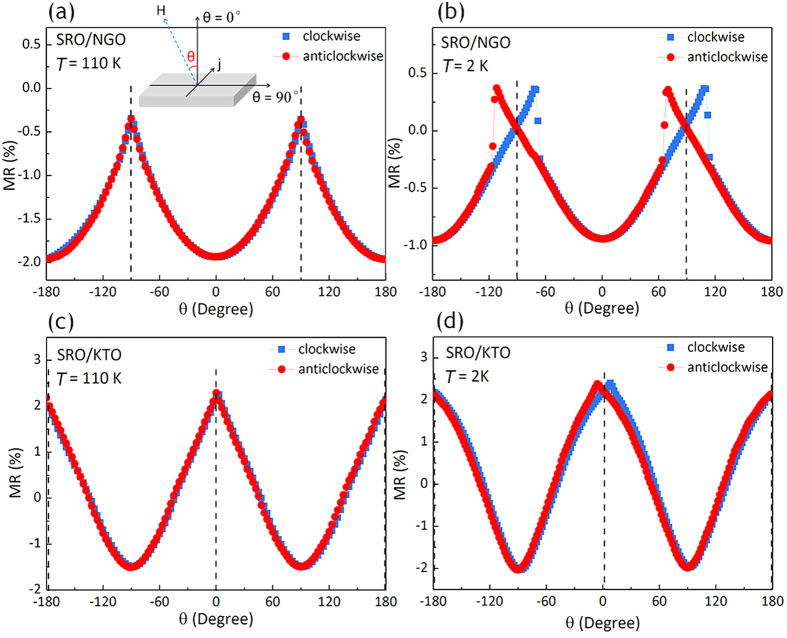
Field-angle dependence of MR for the SRO film on (**a**), (**b**) NGO substrate and (**c**), (**d**) KTO substrate. The measurement was taken at (**a**), (**c**) 110 K and (**b**), (**d**) 2 K. The definition of the field angle θ was shown as inset in 5(**a**).

The inferred anisotropic physical properties from the hysteresis loops of magnetization and MR can be further confirmed by the field angle-dependent electrical transport measurement. Figure 6(a–d) shows the angle-dependent MR by rotating the field in the (100) plane of NGO and KTO respectively. A magnetic field of 4T was applied during the electrical transport measurement to keep the magnetization of SRO in a saturated state. The electrical current was kept perpendicular to the magnetic field throughout the measurement. Similar results are obtained when measuring the angular dependence of MR in the (010) plane. The field angle θ is defined as the angle between the applied field and the film normal direction. Note that sharp jumps or peaks would result from magnetization reversal that takes place once the angle between the field and the easy axis exceeds 90°. For SRO film grown on NGO substrate, peaks are observed at θ =  ±90° when measurement was taken at 110 K (Fig. 6(a)), suggesting the perpendicular magnetic anisotropy of this tetragonal SRO film. For measurement taken at 2 K (Fig. 6(b)), clear peaks are observed with the hysteresis in the clockwise and anticlockwise field rotation after every 180° in θ, revealing the strong magnetic anisotropy of the film. The centre of the hysteresis are seen at θ =  ±90°, revealing that the easy axis of SRO/NGO is along the film normal direction which is the case for tetragonal SRO phase^8^,^17^. For SRO film deposited on KTO substrate, on the contrary, the peaks are found at θ = 0° and 180° (see Fig. 6(c,d)), indicating that the magnetic moment of the SRO/KTO film is confined in the film plane.

**Figure 7:**
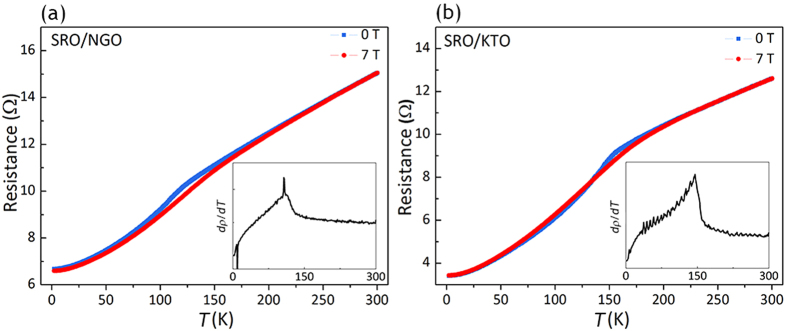
Temperature dependence of resistance for the grown SRO films on (**a**) NGO substrate and (**b**) KTO substrate. Blue curves and red curves were obtained with the applied magnetic field of 0 and 7 Tesla respectively. Insets: temperature derivative of resistivity *dρ/dT* for films on NGO and KTO substrate.

In addition to the magnetic anisotropy investigation of SRO films, the temperature dependence of resistance was shown in Fig. 7(a,b) for the film on NGO and KTO respectively. Singularity was observed in the temperature derivative of resistivity *dρ*/*dT* (insets), indicating the Curie point of each film. It turns out that the SRO/NGO film has a much reduced *T*_c_ ~ 110 K which accords with the tetragonal SRO phase^10^ whilst SRO/KTO film has a *T*_c_ value of ~150 K that is closer to the bulk value of 160 K^16^.

Based on the above results, the tetragonal SRO phase stabilized by moderate compressive strain (e.g. on NGO substrate) has a perpendicular magnetic anisotropy and a reduced *T*_c_ while the tensilely strained SRO film has the characteristic of bulk SRO phase. Therefore it is natural to ask why the compressive strain results in the coherent growth of tetragonal phase, whilst the tensile strain of almost the same amplitude leads to a relaxed bulk-like phase. To understand this the misfit relaxation mechanism was considered. In perovskites with rigid octahedra such as SRO material, octahedral rotation (altered Ru-O-Ru angle) is energetically favorable than octahedral deformation (change in Ru-O bond lengths) to accommodate the misfit strain. Generally speaking, the octahedral tilting about the out-of-plane direction is enhanced under compressive strain (Fig. 1(a)) while it is weakened in response to tensile strain (Fig. 1(c)). For instance, the octahedral tilting about the out-of-plane direction is increased whereby the misfit strain of SRO/NGO is released. For SRO film on KTO substrate however, the unit cell length of KTO (3.99 Å) is larger than the length of fully stretched Ru-O-Ru bond (with a bond angle of 180°) at 3.97 Å. That is to say, octahedral rotation alone is not able to accommodate such a lattice mismatch. Octahedral deformation, on the other hand, is very energetically costly in SRO. As a result, misfit dislocations, another strain relaxation mechanism, are produced in SRO/KTO that led to the relaxed bulk-like SRO phase finally.

In Summary, we have successfully manipulated the octahedral rotation pattern in SRO films by employing compressive and tensile strain. We found that the film under −1.7% compressive strain has the rotation pattern of tetragonal phase **a**^**0**^**a**^**0**^**c**^**−**^ while those under +1.5% tensile strain has the rotation pattern of the bulk-like orthorhombic phase **a**^**−**^**a**^**−**^**c**^**+**^. The physical properties of these films vary according to the octahedral rotation pattern. The compressively-stabilized tetragonal SRO phase exhibits a perpendicular magnetic anisotropy and a reduced Curie temperature, while the orthorhombic SRO film under tensile strain has the magnetic anisotropy and Curie temperature close to that of the bulk. The strain dependent magnetic anisotropy and Curie temperature is due to the diverse response of octahedral behavior to different strains. Our results suggest the significance of strain engineered octahedral architecture in producing novel electronic and magnetic properties. Special attention should be paid to the selection of substrates for the growth of perovskite thin films with desired octahedral rotations and physical properties.

## Methods

SRO epitaxial films were deposited on NGO(110) and KTO (001) substrates by pulsed laser deposition (PLD) with KrF excimer laser focused on stoichiometric ceramic SRO target. The KrF excimer laser had a laser spot density of 1.2 J/cm^2^ and a spot area of 2.5 mm × 2 mm at the target. The film thickness was kept around 60 nm. During deposition, the substrate temperature was kept at 450 °C. The oxygen partial pressure was kept at 100 mTorr. The structural characterization of the grown films was carried out by XRD as well as TEM. The Huber four circle diffractometer system 90000-0216/0 was used at the x-ray development and demonstration (XDD) beam line of Singapore Synchrotron Light Source (SSLS) and the Huber 5021 4 circle +2 circle system was used at the BL14B1 beam line of Shanghai Synchrotron Radiation Facility (SSRF). The cross-sectional TEM samples were prepared by a focused ion beans (FIB) (DA300, FEI) and the TEM observations were performed at room temperature using Tecnai X-TWIN TEM system. The octahedral rotation pattern was determined from the presence or absence of weak ‘extra’ super-reflections (often called half-integer reflection). The magnetic properties of the films were characterized by physical property measurement system (PPMS) equipped with a rotator as well as by the superconducting quantum interference device (SQUID).

## Additional Information

**How to cite this article**: Lu, W. *et al*. Strain Engineering of Octahedral Rotations and Physical Properties of SrRuO_3_ Films. *Sci. Rep.* **5**, 10245; doi: 10.1038/srep10245 (2015).
